# Supplementary material for: Association of Hypernatremia with Immune Profiles and Clinical Outcomes in Adult Intensive Care Unit Patients with Sepsis
Source: Biomedicines. 2022 Sep 14;10(9):2285. doi: 10.3390/biomedicines10092285 (PMC9496274; doi:10.3390/biomedicines10092285)

Association of Hypernatremia with Immune Profiles and Clinical Outcomes in Adult Intensive Care Unit Patients with Sepsis

Supplementary Materials

Table S1. Baseline plasma cytokine levels in patients with and without dysnatremia on admission.

Cytokine	Hyponatremia (n=65)	Eunatremia (n= 80)	Hypernatremia (n=15)	p-value
IL-6 (pg/uL)	64.5 (87.6)	48.3 (145.2)	82.6 (334.1)	0.751
IL-10 (pg/ul)	15.6 (65.4)	23.6 (60.5)	46.7 (203.3)	0.410
G-CSF (pg/ul)	77.0 (188.7)	64.1 (119.3)	73.9 (416.7)	0.805
TNF-α (pg/ul)	43.3 (48.8)	29.1 (48.9)	40.7 (53.5)	0.241

Abbreviations: G-CSF, granulocyte colony-stimulating factor; IL, interleukin; TNF, tumor necrosis factor; IQR, interquartile range.

All data are presented as medians (IQRs).

Table S2. First 3 days of fluid accumulation between patients in each group

	Hyponatremia	Eunatremia	Hypernatremia	p-value
**Day 1**				
Input	1703.7(1400.6)	1735.8(1302.6)	2034.2(1230.7)	0.066
Input-Ouput	597.5(1381.2)	473.9(1351.4)	810.0(1454.6)	0.560
**Day 2**				
Input	2470.2(1412.1)	2408.0(1402.9)	2464.9(1415.8)	0.879
Input-Ouput	899.9(1451.7)	674.3(1458.9)	1081.6(1587.9)	0.098
**Day 3**				
Input	2260.5(1310.3)	2191.6(1389.8)	2299.7(1395.4)	0.451
Input-Ouput	484.2(1392.7)	465.6(1220.3)	760.5(1181.9)	0.097

	Sustained eunatremia	Acquired hypernatremia	p-value
**Day 1**			
Input	1717.1(1287.3)	1933.4(1631.2)	0.482
Input-Ouput	431.7(1353.5)	449.7(1539.6)	0.506
**Day 2**			
Input	2494.5(1441.3)	2619.7(1408.4)	0.266
Input-Ouput	616.0(1460.9)	755.6(1679.1)	0.456
**Day 3**			
Input	2322.5(1441.1)	2459.9(1202.7)	0.177
Input-Ouput	512.4(1177.9)	462.7(1539.7)	0.778

All data are presented as mean (standard deviation); the unit of data is milliliters.

Figure S1. Kaplan–Meier survival curve of patients with sustained eunatremia, sustained hypernatremia, progression from eunatremia to hypernatremia, or regression from hypernatremia to eunatremia.

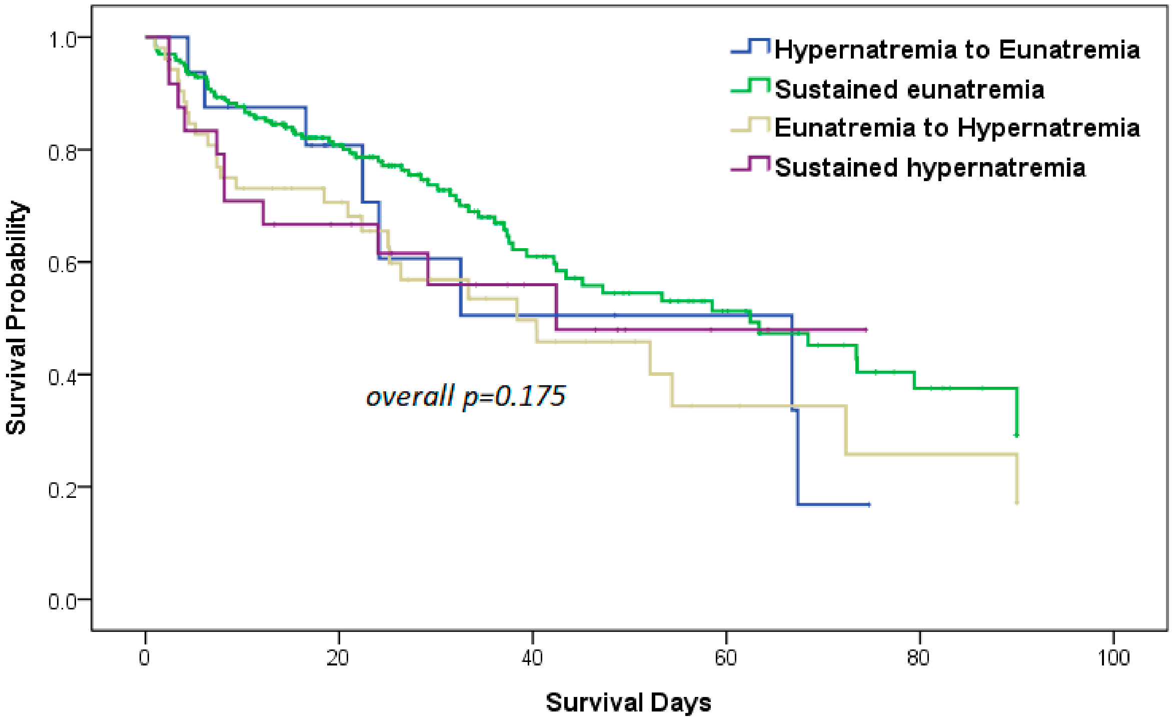

Supplement: Supplementary file 1 [file biomedicines-10-02285-s001.zip › biomedicines-1849703-supplementary.pdf]
